# Comparison of case-based learning combined with Rain Classroom teaching and traditional method in complete denture course for undergraduate interns

**DOI:** 10.1186/s12909-022-03678-z

**Published:** 2022-08-09

**Authors:** Xueling Li, Yanshan Li, Xiaolan Li, Xiaodan Chen, Guihong Yang, Ling Yang

**Affiliations:** Hospital of Stomatology, Guanghua School of Stomatology, Guangdong Provincial Key Laboratory of Stomatology, Sun Yat-Sen University, 56 Lingyuan Road West, Guangzhou, 510055 Guangdong China

**Keywords:** Case-based learning, Rain Classroom teaching, Interns, Complete denture, Clinical practice

## Abstract

**Background:**

Complete denture, as an important restoration method for edentulism, is difficult to study for beginners, especially in linking the theory with clinical practice.

**Objective:**

This study was aimed to compare the teaching effects between case-based learning combined with Rain Classroom teaching and traditional lecture method in the clinical course of complete denture prosthesis for undergraduate interns.

**Methods:**

In a course called “Problems and treatment strategies of complete denture after wearing”, interns were divided into two groups: one for traditional lecture-based teaching with PowerPoint slideshow (the control group, n = 28); and the other for case-based learning combined with Rain Classroom teaching, which published information before class, discussed specific clinic cases in class and got real-time interns’ feedback via WeChat (the test group, n = 22). Both groups received the same exam and questionnaire survey after class. The Q&A participation of interns in class, theoretical test scores and questionnaire survey responses were used to evaluate the teaching effects. An independent sample t-test and the chi-square test or Fisher’s exact test were used for statistical analysis in this study.

**Results:**

The Q&A participation of interns in the test group was much better than that of the control group. The average score on the theoretical test after class in the test group (72.14 ± 12.24) was significantly higher than that in the control group (61.29 ± 20.12) (*P* < 0.05). In the test group, 94.54% (21/22) of the interns preferred the new teaching mode.

**Conclusion:**

Case-based learning combined with Rain Classroom teaching is helpful to enliven the classroom atmosphere, inspire studying enthusiasm, and achieve a good learning effect in both theory and clinical practice related to complete denture prosthesis.

## Background

Edentulism is very common worldwide in the elderly population over the age of 65 [1]. According to the 4th National Oral Health Survey in China, the number of edentulous patients increased significantly with age, and the proportion of edentulous patients in elderly people aged 65–74 was 4.5% [2], with a total population of more than 21 million. The increase in prosthetic restorations for elderly individuals due to longer life expectancy means that the demand for prosthodontic treatment will increase in the next few decades due to a higher frequency of edentulism, even in countries with a high standard of dental health care [3]. Because of the high price of complete implant dentures and high requirements for general health and local alveolar bone conditions, a large proportion of edentulous patients still choose traditional complete dentures [4, 5]. Therefore, complete denture prosthesis is one of the most important techniques that stomatology students must master, and education on complete dentures is a necessary part of the curriculum for dental undergraduates in China. In our dental school, 20 credit hours for lectures in class (from the initial clinical reception to problems and treatment strategies after denture wearing) and 36 credit hours for practical training in lab are arranged for complete denture prosthesis for undergraduates. Usually, the teachers present their lectures via PowerPoint slideshow full of clinical photos and videos and give interns denture manufacturing process training step by step. After that, students have 16-week practice in clinic as interns. Although careful preparation and patient instruction of teachers, the teaching effect of complete denture prosthesis is not satisfactory either in scores on the prosthodontics theoretical test nor in performance of clinic practice. The theory related to complete dentures is abstract and unintelligible, and the clinical procedures are complex and difficult to understand and grasp, especially for interns with little clinical experience [6, 7]. Most interns have no confidence in clinical reception of edentulous patients. Moreover, due to pain, poor retention or other reasons, most patients need follow-up adjustment several times after restoration [3], which is more difficult for interns. They have a low ability to deal with these problems effectively after internship. To improve interns’ clinical skills related to complete dentures and to help them better deal with a series of problems after restoration, a clinical course called “Problems and treatment strategies of complete denture after wearing” was established during the internship. Traditional lecture teaching methods, which are generally teacher-centred, encounter serious challenges since they usually fail to promote students' learning initiatives and to develop their learning abilities [8, 9]. The classroom atmosphere of traditional teaching is inactive, and most students accept knowledge passively [8], which leads to an unsatisfactory teaching effect. On the other hand, case-based learning (CBL), which is widely used in medical education, is an active learning strategy that focuses on students as the centre of the learning environment [8] by allowing students to study human cases, improving students’ learning interest, stimulating self-guided learning and activating the classroom. Rain Classroom system is another teaching solution to improve the teaching quality, which is a new intelligent jointly launched by Tsinghua University and Xuetang online. It is a software plug-in that integrates PowerPoint and WeChat. It aims to connect teachers’ and students’ intelligent terminals; offer students new experiences in every teaching process before, during and after class; energize teaching and learning to the greatest extent; and promote teaching reform [9].

To stimulate the learning enthusiasm of interns and cultivate their ability to analyse and solve clinical problems related to complete dentures, CBL combined with Rain Classroom teaching was applied during the newly established clinical course. By comparing the classroom performance, knowledge mastery and evaluation of teaching effect of undergraduate interns under the two teaching modes, this study aimed to explore the effect of CBL combined with Rain Classroom in clinical course on complete denture prosthesis.

## Methods

### Clinical course and participants

The clinical course called "Problems and treatment strategies of complete denture after wearing" was established at the last month of the internship in prosthodontics department, and it was taught by the same senior teacher using two different teaching methods. One was the traditional lecture methods, and the other was CBL combined with Rain Classroom teaching. The teaching plan about the two teaching methods was told to the undergraduate interns of the 2016 grade from Guanghua School of Stomatology, Sun Yat-sen University in advance. Before their clinical practice, all interns will draw lots to determine the practice sequence in prosthodontics, dental endodontics and maxillofacial surgery. The interns in the first-round practicing in the prosthodontics department were designated as the test group, and those in the second round were designated as the control group. Fifty undergraduate interns were recruited as participants. All interns who refused to participate were excluded from the study. This study was approved by the Medical Ethics Committee of Hospital of Stomatology, Sun Yat-sen University (Approval no. KQEC-2021–60-01). CBL combined with Rain Classroom teaching was applied in the test group, with a total of 22 interns (9 males and 13 females), while the traditional teaching method was used in the control group, with a total of 28 interns (11 males and 17 females). There was no significant difference between the two groups in terms of age, gender or scores on the prosthodontics theoretical test in the second semester of senior year (*p* > 0.05).

### Teaching method

In the test group, the interns were informed to visit the official account platform of Rain Classroom three days before class. The teacher uploaded and published micro-courseware and a typical case complaining about some problems after complete denture prosthesis via Rain Classroom system. Then, the teacher posted four relevant questions and required the students to discuss them in subgroups, such as the possible reasons for pain, cheek biting and poor retention for the complete dentures. The teacher was able to view whether the students had previewed the material through Rain Classroom. Before teaching, the teacher obtained the patient’s permission to allow his data for teaching. At the beginning of the class, five single-choice questions were released through Rain Classroom. This was not only to test the effect of preview and the interns’ prior knowledge on complete denture, but also to make them familiar with the operation of Rain Classroom. Next, a representative of each subgroup made a 3–5-min report of the question assigned. Then, the teacher combined the key points of each question and analysed in-depth the causes and solutions of the main problems of the complete denture after wearing. Five single-choice questions about this case and the key points were then interspersed through Rain Classroom. The interns were asked to answer through the Rain classroom and the answers could be immediately presented in the form of charts in the class.

The traditional lecture teaching method was adopted in the control group. Three days before class, the interns received a task to preview the textbook and 4 questions similar to the test group. In class, multimedia courseware was used as usual with many clinical pictures and some videos. Combined with clinical case, the teacher analysed the causes of various clinical problems after complete denture prosthesis and then presented the corresponding solutions one by one. The whole class was mainly taught by the teacher. To strengthen classroom interaction, the same five single-choice questions were also asked during class, which were answered voluntarily or by roll call. In both the test and control groups, the key points were summarized using a mind map before the end of the course. Then, the two groups took the same test via a professional online platform for questionnaire, survey, and examination named as “wenjuanxing”, including 12 single-choice questions (objective questions, 5 points per question, 60 points totally) and 2 essay questions (subjective questions, 20 points per question, 40 points totally) immediately after the class. The single-choice questions tested interns' grasp of basic knowledge points, while essay questions need interns applying their knowledge to realize the problems, to analyse possible reasons and to give specific solutions under certain circumstances in clinic.

In addition, a self-designed questionnaire was distributed to the interns through WeChat to investigate their feelings and perceptions about the learning burden, teaching content, teaching method and teaching result. Each item was scores according five levels: strongly agree, agree, neutral, disagree and strongly disagree. The score ranged from 1–5. The lower the score was, the higher the interns’ acceptance of the teaching mode.

**Fig. 1:**
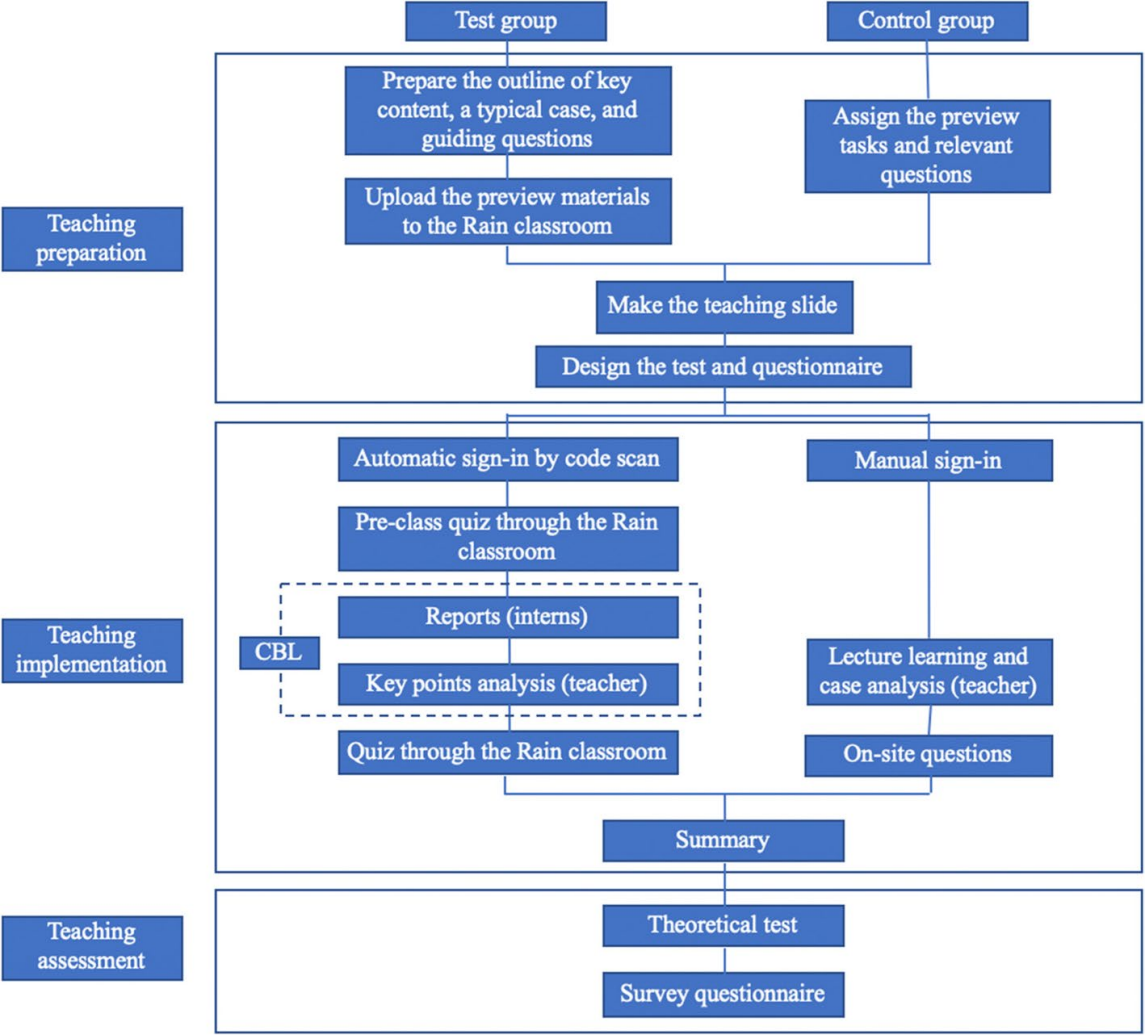
Teaching flow chart

The specific implementation process is shown in Fig. 1.

### Statistical indicators

The preview time, derived from the Rain Classroom platform in the test group and the questionnaire survey in the control group, represented the learning burden. The Q&A participation of interns in class was used as a descriptive indicator to reflect the activity of the classroom. The theoretical test score was composed of objective and subjective scores. The objective questions were scored automatically by the system, and the subjective questions were scored by the same teacher. The overall impressions on the teacher’s teaching, teaching content, teaching method and teaching results from the questionnaire survey were used to evaluate the teaching effects.

### Statistical analysis

All statistical analyses were carried out using SPSS 26.0 software. A single-sample K-S test was used to verify the normal distribution of measurement data, and an independent sample t-test was used for normally distributed samples. The chi-square test or Fisher’s exact test was used to determine if there was a significant difference between the groups for categorical data. A *P*-value less than 0.05 was considered significant.

## Results

### Preview time

All interns’ preview time was less than 30 min, and more than half of the interns’ preview time was no more than 10 min. There was no significant difference in preview time between the two groups (*P* > 0.05) (Table 1).

**Table 1:** Comparison of preview time between the two groups [n(%)]

Preview time (min)	Test group	Control group	Fisher exact probability	
			*χ*^2^value	*P-*value
≤ 10	13(59.09%)	14(50.00%)	3.86	0.153
10–20	8(36.36%)	7(25.00%)		
20–30	1(4.55%)	7(25.00%)		
total	22	28		

### Class activity

Class activity was reflected by the Q&A participation of interns. In the test group, most interns followed the teacher's rhythm and answered the questions through Rain classroom. Finally, 91 answers were collected in total for the five questions. While in the control group, as no volunteer stood up to answer the same questions in class, 5 interns were selected by the teacher to answer these questions, namely 5 answers were collected. These data showed the class activity of the test group was much better than that of the control group.

### Comparison of theoretical test scores

The scores of the objective questions and the total score in the test group were significantly higher than those in the control group (*P* < 0.05). There was no significant difference in the scores of the subjective questions between the two groups (*P* > 0.05) (Table 2).

**Table 2:** Comparison of theoretical test scores between the two groups  ± *s*, points)

	Test group (*n* = 22)	Control group (*n* = 28)	*t* value	*P-*value
Score of objective questions	45.45 ± 5.32	39.11 ± 12.62	2.40	0.021*
Score of subjective questions	26.68 ± 9.89	22.18 ± 10.03	1.59	0.119
Total score	72.14 ± 12.24	61.29 ± 20.12	2.22	0.031*

^*^There was a significant difference between the two groups (*P* < 0.05)

### Comparison of teaching effect

According to the questionnaire survey, both groups of interns had a high overall impression of teachers’ teaching, and there was no significant difference between the groups (*P* > 0.05) (Table 3).

**Table 3:** Comparison of the overall impression of teachers’ teaching [n(%)]

Overall impression score	Test group	Control group	Fisher exact probability	
			*χ*^*2*^ value	*P*-value
≥ 90	18(81.82%)	23(82.14%)	0.92	1.000
80–89	4(18.18%)	4(14.29%)		
70–79	0(0.00%)	1(3.57%)		
total	22	28		

All interns in both groups believed that the course content was closely related to clinical practice and had strong practicability. They also agreed that the teacher focused on the key points and explained the difficulties thoroughly. There was no significant difference in teaching content scores between the two groups (*P* > 0.05). In terms of teaching methods, both groups agreed that the teacher could apply modern educational technology in teaching effectively. The heuristic and discussion teaching scores of the test group were significantly lower than those of the control group (*P* < 0.05). At the end of the questionnaire, 94.54% (21/22) of the interns in the test group believed that CBL combined with Rain Classroom teaching was better than traditional teaching. They thought that the classroom atmosphere of the former was more active than that of the latter, and their scores were significantly lower than those in the control group *(P* < 0.05) (Table 4).

**Table 4:** Comparison of teaching effects between the two groups

		Test group	Control group	*P* value
Teaching content	Relates to clinical practice	1.36 ± 0.49	1.43 ± 0.50	0.650
	Highlights the key points and explains the difficulties thoroughly	1.36 ± 0.58	1.43 ± 0.50	0.674
Teaching method	Uses heuristic and discussion teaching	1.41 ± 0.50	1.75 ± 0.65	0.047*
	Uses modern educational technology	1.41 ± 0.85	1.64 ± 0.78	0.318
Teaching result	Helps improve classroom efficiency	1.50 ± 0.67	1.68 ± 0.55	0.306
	Helps cultivate self-study ability	1.64 ± 0.79	1.46 ± 0.58	0.396
	Helps absorb knowledge rapidly	1.36 ± 0.58	1.32 ± 0.48	0.779
	Creates an active classroom atmosphere	1.45 ± 0.51	1.93 ± 0.81	0.021*
	Stimulates interest and initiative in learning	1.50 ± 0.67	1.64 ± 0.68	0.462
	Helps cultivate speculative ability	1.55 ± 0.60	1.46 + 0.51	0.606

^*^There was a significant difference between the two groups (*P* < 0.05)

## Discussion

### Practicality of the course

Implant dentures are regarded as the best restoration technique because they can better restore and reconstruct the oral and maxillofacial function of edentulous patients [4]. Due to general health, local alveolar bone condition and economic status, many patients tend to choose traditional complete denture prosthesis instead of implant dentures. Therefore, complete denture will continue to be the most economical and common treatment to reconstruct oral and maxillofacial function in edentulous patients [4]. The procedure of complete denture prosthesis is precise and complex. Furthermore, a series of problems, such as pain or poor retention, often occur after restoration for various reasons. Dentures also need to be maintained due to changes in soft and hard tissue after wearing for a period of time [3]. Because of a lack of clinical experience, interns usually fail to integrate theory with the actual situation of patients and lack the ability to analyse and solve all kinds of clinical problems. Many students believe that increasing their clinical experience would be most beneficial in increasing their confidence levels [10]. Hence, the course "Problems and treatment strategies of complete denture after wearing" was established during clinical practice to train the interns in clinical reasoning and skill, which could increase their confidence in solving practical clinical problems. The course was popular with interns. Both groups of interns in this study reported that the teaching content was closely related to clinical practice and had strong practicability.

### Main role of Rain Classroom

Rain Classroom integrates complex information technology into PowerPoint and WeChat and establishes a communication bridge between the pre-class preview and classroom teaching so that classroom interaction will never go offline. Rain Classroom scientifically covers every teaching process before, during and after class [11]. All kinds of learning materials, courseware, exercises, assessments, and references can be delivered to students' mobile devices. Then, teachers and students can communicate and give feedback before or after class [9].

During the class, Rain Classroom is proved to upgrade teaching quality by getting instant feedback from all students via statistical results display or Danmaku [12], compared to the given traditional PowerPoint slideshow lessons. In this study, real-time answers and bullet screen interactions in the classroom provide a perfect solution for interactions between teachers and students. After the students answer questions in class, the teacher and students can see the results immediately because all the answers can be (anonymously) displayed on the screen in form of histogram, and they can clearly determine their own problems. Moreover, teachers can quickly grasp the learning situation to adjust the teaching focus and improve the teaching quality.

How to increase the students' interest in learning and how to active the classroom atmosphere are concerned by many teachers [13, 14]. An active classroom atmosphere helps improve students' concentration and performance and makes them transition from passive learning to active learning. For students, Rain classroom motivate learning enthusiasm, as well as improve learning outcomes [12]. A 10-week Rain Classroom teaching experiment among students majoring in clinical medicine was performed, and over 90% students believed that they could easily access teaching resources at any time via their mobile phones, conduct effective exchange with teachers and understand their shortage of the overall lesson through Rain Classroom [15]. Another study about “computer-aided landscape design” suggested that students who participated in the Rain Classroom scored significantly higher by an average of > 3 percentage points than that in the traditional lessons [16].

In this study, we found that the in-class test through Rain Classroom enhanced the interaction between teachers and interns and significantly improved classroom activity to maintain the interns' focus on learning. Through a questionnaire survey, we found that the interns showed a good reaction to the classroom atmosphere. Thus, Rain Classroom teaching can strengthen the interaction between teachers and students, especially for students who are not good at communication, because they can communicate with teachers and discuss problems through Rain Classroom.

Moreover, instead of manual check-ins, the code scanning check-in feature of Rain Classroom is convenient and effective. Teachers can clearly know the attendance rate of students, which can reduce the truancy rate to some extent [17].

### Role of CBL in clinical practice

CBL is defined as a case-based education method that is based on the analysis of medical records with the aim of restoring the real clinical scene and prompting students to develop new area of learning [18, 19]. CBL is used in a variety of medical fields using human cases to impart relevance and aid in connecting theory to practice. It encourages students to use techniques that help them integrate, synthesize, and apply newly learned information to a broader context, both to help them see the value of what they are learning and to foster critical-thinking skills [20]. Many studies have shown that this teaching method can improve students' interest and effect in learning [8, 21, 22]. Rhodes A suggested that compared with traditional lecture learning, CBL does not guarantee improved learning gains because the extraneous load potentially presented by CBL might overwhelm the cognitive abilities of inexperienced students [23]. However, this depends on students' personality characteristics and level of cognitive abilities. Chinese students have long been accustomed to traditional lecture teaching and are not good at expressing their views openly in class [24]. Historically, most students have not liked to ask questions or express their opinions in class.

CBL is often used in medical teaching in China [8, 19]. In this study, the interns were asked to discuss four questions of the case in groups. They showed great enthusiasm about the case and expressed their group opinions respectively during class.

### Teaching effect

In this study, the interns in the test group stated that the classroom atmosphere was active, and they did not easily become distracted, so they could keep up with the teacher's rhythm very well. The result of the theoretical test after class in the test group was significantly higher than that in the control group, indicating that interns with a high classroom involvement had a better learning effect. The scores of objective questions in the test group were significantly higher than those in the control group. This might be because the objective questions mainly assessed the interns' mastery of knowledge points. That is, the interns in the test group absorbed and accurately mastered the knowledge points in a short time. The classroom teaching efficiency in the control group was not as good as that in the test group. However, there was no significant difference in the scores of the subjective questions between the two groups. It might be that the subjective questions mainly assessed the interns' comprehensive application ability of knowledge, which required a long time to cultivate, rather than being immediately acquired.

### Requirements for teachers

The purpose of education is not to obtain knowledge but to develop one's potential ability and to obtain wisdom [9]. At this level of education, the most important task of teachers is not only to teach students existing knowledge but also to constantly guide them in what to learn and how to learn effectively. Therefore, the role of teachers has changed from a knowledge imparter to a guide [9, 25].

Rain Classroom is an effective tool designed to help teachers to achieve this educational requirement. Information publishing before class, the real-time feedback and multi-screen interaction in class, as well as the reviewing after class of Rain Classroom, guide students to follow teachers’ steps to learn both knowledge and thinking methods [26].

CBL combined with Rain Classroom imposes higher requirements for teachers [9, 25]. In addition to making teaching slides, teachers also need to prepare preview instructions, including massive open online courses (MOOC) videos, typical cases and some guiding questions, and prepare in-class tests before teaching. During class, teachers should analyse the learning situation according to the students' feedback and quickly adjust the teaching plan. Moreover, teachers should not only pay attention to introverted and shy students but also properly control hyperactive students, maintain an orderly classroom and ensure the effectiveness of the classroom environment. After class, teachers should respond to students' questions if they have any. Therefore, teachers with a strong learning ability, sense of responsibility and the patience and courage to try new things can do a good job in teaching under this new mode.

### Limitation of this teaching method

Some limitations about this teaching method should be discussed here. 

Firstly, as mentioned above, CBL combined with Rain Classroom throws higher requirements on teachers and increase the teaching burden. In view of this problem, two suggestions are put forward. On one hand, a teaching team, well collaborated and closely cooperated, should be established. Members share the teaching jobs, such as clinic data collection or questionnaire preparation, and the organizer integrate the resources. On the other hand, teaching contents should be well selected and suitable for such teaching methods. Cases are typical with appropriate difficulty for study and discuss.

Secondly, Rain Classroom teaching depends on the stability of the network and is affected by teaching equipment. This is another weak point. So, before you perform your teaching via Rain Classroom, Classroom equipment and network should be guaranteed. Otherwise, real-time feedback won't work, and make a mess in the class.

Thirdly, too much feedback from the students via Danmaku may interference the class. Thus, the teacher should control the pace of lectures and allow interaction with students at appropriate time.

## Conclusions

It is necessary to establish clinically relevant courses during internships. Rain Classroom can efficiently connect each teaching process before, during and after class. CBL combined with Rain Classroom teaching is helpful to enliven the classroom atmosphere, inspire studying enthusiasm, and achieve good learning effects in both theory and clinical practice related to complete denture prosthesis.

## Data Availability

The data described in this article can be freely and openly accessed at Harvard Dataverse: https://doi.org/10.7910/DVN/54R2MJ

## Abbreviations

CBL: Case-based learning
MOOC: Massive open online courses
